# Genome-wide identification and characterization of long non-coding RNAs conferring resistance to *Colletotrichum gloeosporioides* in walnut (*Juglans regia*)

**DOI:** 10.1186/s12864-020-07310-6

**Published:** 2021-01-06

**Authors:** Shan Feng, Hongcheng Fang, Xia Liu, Yuhui Dong, Qingpeng Wang, Ke Qiang Yang

**Affiliations:** 1 College of Forestry, Shandong Agricultural University, Tai’an, 271018 Shandong Province China; 2 State Forestry and Grassland Administration Key Laboratory of Silviculture in the Downstream Areas of the Yellow River, Tai’an, 271018 Shandong Province China; 3 Shandong Taishan Forest Ecosystem Research Station, Tai’an, 271018 Shandong Province China; 4 Department of Science and Technology, Qingdao Agricultural University, Qingdao, 266109 Shandong Province China

**Keywords:** Walnut (*Juglans regia* L.), *Colletotrichum gloeosporioides* (Penz.) Penz. And Sacc., lncRNA, WGCNA

## Abstract

**Background:**

Walnut anthracnose caused by *Colletotrichum gloeosporioides* (Penz.) Penz. and Sacc. is an important walnut production problem in China. Although the long non-coding RNAs (lncRNAs) are important for plant disease resistance, the molecular mechanisms underlying resistance to *C. gloeosporioides* in walnut remain poorly understood.

**Results:**

The anthracnose-resistant F26 fruits from the B26 clone and the anthracnose-susceptible F423 fruits from the 4–23 clone of walnut were used as the test materials. Specifically, we performed a comparative transcriptome analysis of F26 and F423 fruit bracts to identify differentially expressed LncRNAs (DELs) at five time-points (tissues at 0 hpi, pathological tissues at 24 hpi, 48 hpi, 72 hpi, and distal uninoculated tissues at 120 hpi). Compared with F423, a total of 14,525 DELs were identified, including 10,645 upregulated lncRNAs and 3846 downregulated lncRNAs in F26. The number of upregulated lncRNAs in F26 compared to in F423 was significantly higher at the early stages of *C. gloeosporioides* infection. A total of 5 modules related to disease resistance were screened by WGCNA and the target genes of lncRNAs were obtained. Bioinformatic analysis showed that the target genes of upregulated lncRNAs were enriched in immune-related processes during the infection of *C. gloeosporioides*, such as activation of innate immune response, defense response to bacterium, incompatible interaction and immune system process, and enriched in plant hormone signal transduction, phenylpropanoid biosynthesis and other pathways. And 124 known target genes for 96 hub lncRNAs were predicted, including 10 known resistance genes. The expression of 5 lncRNAs and 5 target genes was confirmed by qPCR, which was consistent with the RNA-seq data.

**Conclusions:**

The results of this study provide the basis for future functional characterizations of lncRNAs regarding the *C. gloeosporioides* resistance of walnut fruit bracts.

**Supplementary Information:**

The online version contains supplementary material available at 10.1186/s12864-020-07310-6.

## Background

Walnut (*Juglans regia* L.) is a diploid tree species (2n = 32), with approximately 667 Mb per 1C genome and an N50 size of 464,955 (based on a genome size of 606 Mbp) [1]. It is an ecologically important ‘woody oil’ tree species worldwide [2], and its kernel is a rich source of nutrients with health benefits for humans [3]. The peptides extracted from walnut seeds have antioxidant and anticancer activities and have the protective effects on the oxidative damage induced by H_2_O_2_ [4]. Recent advances in biotechnology and genomics show potential to accelerate walnut breeding, such as gamma-irradiated pollen inducing haploid walnut plants [5], constructing the novel Axiom *J. regia* 700 K SNP array [6], and combining different assemblies to obtain the optimal version [7]. Walnut anthracnose caused by *Colletotrichum gloeosporioides* (Penz.) Penz. and Sacc can cause leaf scorch or defoliation and fruit gangrene, which is currently the disastrous disease in walnut production [8]. Due to the long incubation period of anthracnose, the concentrated onset time, and the strong outbreak, the use of chemical fungicides is still the main method of disease control [9]. The *C. gloeosporioides* lifestyle transitions associated with the infection of the host include the following three stages: attachment, biotrophy, and necrotrophy [10]. The pathogen of *C. gloeosporioides* in walnut overwinters in the diseased part with mycelium, and begins to move when the temperature reaches 11–15 °C in the following spring [11]. Specifically, the formation of adherent cells is critical for fungal development during the *C. gloeosporioides* infection [12]. In a previous study, LAC2 was revealed to contribute to the formation of adherent cells to enhance the pathogenicity of *C. gloeosporioides* [13]. However, it is unclear how walnuts recognize and resist infections by *C. gloeosporioides*, and the regulatory network of hub and peripheral genes underlying the resistance of walnuts to *C. gloeosporioides* remains uncharacterized. Therefore, elucidating the molecular basis of this resistance mechanism is imperative for the breeding of walnut resistant to *C. gloeosporioides* [8, 14, 15].

Long non-coding RNA (lncRNA) is a type of RNA comprising 200–1,000,000 nt and structural characteristics similar to those of mRNA, but it does not encode a protein [16]. The lncRNAs were initially considered to be the transcription ‘noise’ of protein-coding genes, and were often ignored in transcriptome analyses [17]. However, the continuous development of sequencing technologies and transcriptome analyses has revealed that many lncRNAs in *Arabidopsis thaliana* [18], *Triticum aestivum* [19], *Zea mays* [20], and other plant species are related to stress responses, morphological development, and fruit maturation. For example, a heat-responsive lncRNA (TCONS_00048391) is an eTM for bra-miR164a and may be a competing endogenous RNA (ceRNA) for the target gene *NAC1* (Bra030820), with effects on bra-miR164a expression in Chinese cabbage (*Brassica rapa ssp. chinensis*) [21]. Qin et al. confirmed that the DROUGHT INDUCED lncRNA regulates plant responses to abiotic stress by modulating the expression of a series of stress-responsive genes [22]. In *A. thaliana*, two lncRNAs, COOLAIR and COLDAIR, are associated with FLOWERING LOCUS C and play an crucial role in vernalization [23, 24].

Many recent studies have proved that lncRNAs are important for plant–pathogen interactions. A role for nine hub lncRNAs and 12 target genes in the resistance of *Paulownia tomentosa* to witches’broom was uncovered via a high-throughput sequencing experiment, and their functions were analyzed with an RNA-lncRNA co-expression network model [25]. In tomato (*Solanum lycopersicum*), the lncRNA16397-GRX21 regulatory network reportedly decreases the reactive oxygen species content and cell membrane damage to enhance the resistance to *P. infestans* [26]. Moreover, the involvement of the WRKY1-lncRNA 33,732-*RBOH* module in regulating H_2_O_2_ accumulation and resistance to *P. infestans* was determined based on a comparative transcriptome analysis [27]. In cotton (*Gossypium* spp.), a functional analysis demonstrated that a lack of two hub lncRNAs, GhlncNAT-ANX2 and GhlncNAT-RLP7, enhances seedling resistance to *Verticillium dahliae* and *Botrytis cinerea*, possibly because of the associated upregulated expression of *LOX1* and *LOX2* [28]. In wheat (*Triticum aestivum* L.), lncRNAs have a tissue-dependent expression pattern that can respond to powdery mildew infections and heat stress [29]. Additionally, four kinds of lncRNAs have important effects on *Puccinia striiformis* infections [30]. However, there are no reports regarding the role of lncRNAs in the walnut fruit resistance to anthracnose.

In this study, Illumina HiSeq 4000 sequencing was used to analyze the disease-resistant (F26) and susceptible (F423) fruit bracts at different *C. gloeosporioides* infection stages. The number and characteristics of lncRNAs were analyzed. Additionally, the hub lncRNAs related to disease resistance were screened and functionally analyzed to predict the role of lncRNAs in walnut fruit bract resistance to anthracnose. To the best of our knowledge, this is the first report on walnut lncRNAs and their biological functions related to fruit bract resistance to *C. gloeosporioides*. Our data may be a useful resource for clarifying the regulatory functions of lncRNAs influencing walnut fruit resistance to *C. gloeosporioides*.

## Results

### Symptoms and physiological changes of walnut fruit infected by *C. gloeosporioide*

**Fig. 1:**
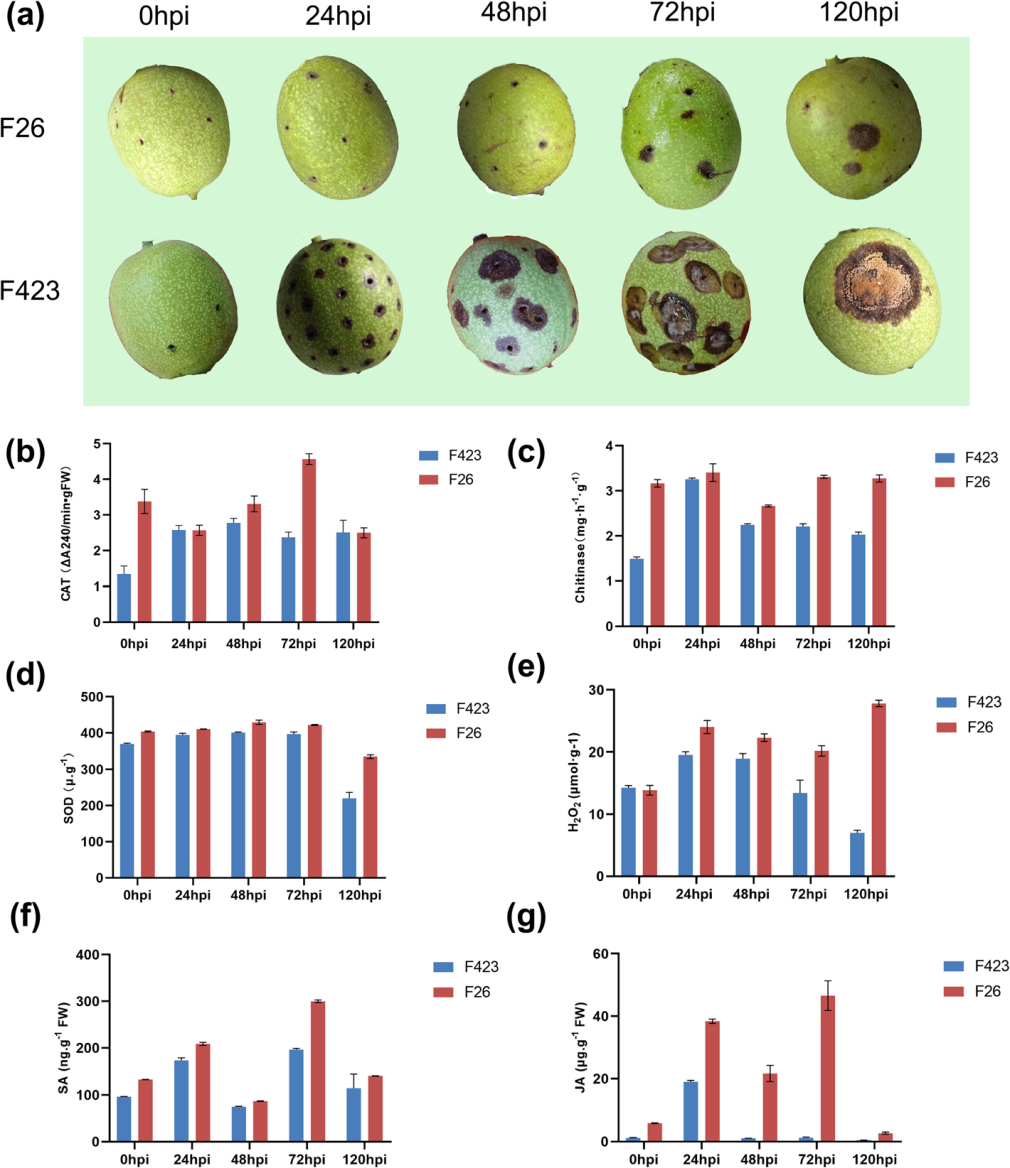
**a** Symptoms of walnut fruit after infection by C. gloeosporioide. **b**-**g** Changes of physiological activity in walnut fruit after infection by C. gloeosporioides. **b** catalase (CAT); **c** Chitinase; **d** superoxide dismutase (SOD); **e** H2O2; **f** salicylic acid (SA); **g** jasmonic acid (JA), respectively

The resistant (F26) and susceptible (F423) fruit bracts were infected by *C.gloeosporioide*, the fruit bracts of F423 showed obvious symptoms at 48 hpi; the disease-resistant fruit F26 at 72 hpi. The susceptible samples showed obvious *C.gloeosporioide* conidial at 120 hpi (Fig. 1a). During the infection, the activities of some enzymes and the content of hormones also changed correspondingly. Compared to the F423, the activities of chitinase, ROS-scavenging enzymes (catalase, CAT and superoxide dismutase, SOD) and the content of H_2_O_2_ in F26 were higher (Fig. 1b-e). The content of salicylic acid (SA) and jasmonic acid (JA) in F26 was significantly higher than that in F423, and reached a peak at 72hpi after infection (Fig. 1f, g).

### Whole genome identification of lncRNAs expressed in walnut fruit bracts

**Fig. 2:**
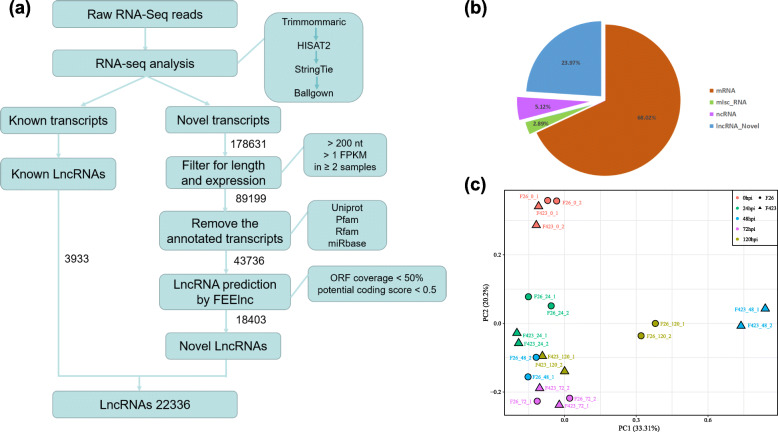
Identification and characterization of long non-coding RNAs (lncRNAs) in walnut. **a** Bioinformatic pipeline for the identification of lncRNAs in walnut. Each step is described in detail in the Materials and Methods section. **b** Proportion of transcripts corresponding to lncRNAs. **c** Patterns of gene expression represented by principal component analysis (PCA) plots of normalized count matrices for walnut fruit bracts

To identify lncRNAs expressed in walnut fruits in response to *C. gloeosporioides*, we constructed 20 cDNA libraries from the anthracnose-resistant and the anthracnose-susceptible walnut fruits at the following five infection stages: tissue at 0 hpi (hours post inoculation), infected tissue at 24, 48, and 72 hpi, and distal uninoculated tissue at 120 hpi (Additional file 1: Table S1). The libraries were sequenced with an Illumina HiSeq 4000 platform. A total of 265.4 Gb clean data were obtained, with an average of 13.27 Gb per library. Approximately 69.7% of the clean reads in all libraries were mapped to the walnut reference genome (Additional file 2: Table S2). The aligned transcripts were assembled, combined, and screened with the FEELnc software to obtain 22,336 lncRNAs (length ≥ 200 nt, ORF coverage < 50%, and potential coding score < 0.5), including 18,403 unknown lncRNAs (23.97%) and 3933 known lncRNAs (5.12%) (Fig. 2a,b). The principal component analyses (PCA) revealed that the results at same infection point were parallel (Fig. 2c).

### Characterization of walnut fruit bract lncRNAs

**Fig. 3:**
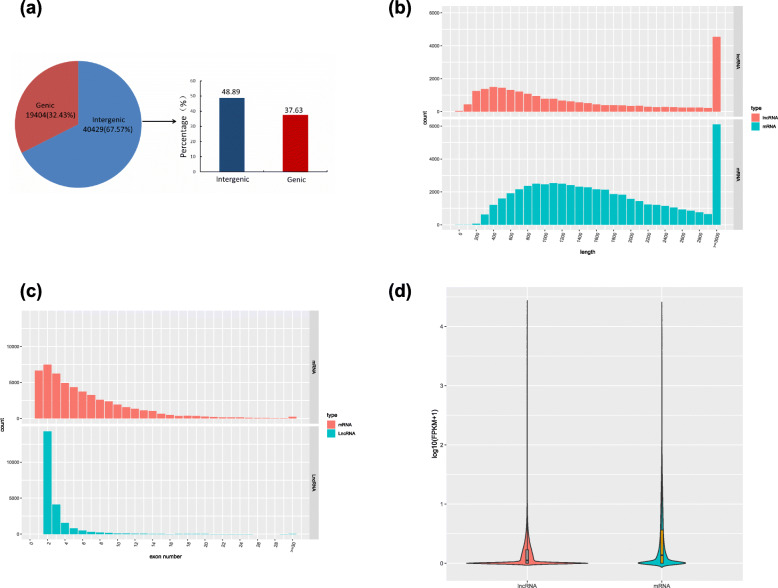
Characteristics of walnut lncRNAs. **a** Proportion of lncRNAs that are located in intergenic and genic regions. **b** Length distribution of 22,336 newly predicted lncRNAs (red) and 58,369 protein-coding transcripts (blue). **c** Distribution of exon numbers in protein-coding genes (red) and lncRNA genes (blue). **d** Expression levels of protein-coding genes and lncRNA genes presented as log_10_ (FPKM + 1) values

A total of 58,369 mRNAs and 22,336 lncRNAs were obtained for the walnut fruit bracts (all samples combined) (Additional file 3: Table S3, Additional file 4:Table S4). The lncRNAs were characterized according to their locations relative to the partner RNA. A total of 40,429 (67.57%) lncRNAs were located in intergenic regions (i.e., only 32.43% genic lncRNAs). Additionally, 19,767 (48.89%) and 7302 (37.63%) of the intergenic lncRNAs and genic lncRNAs were located in the antisense strand, respectively (Fig. 3a) (Additional file 5: Table S5). Most lncRNAs contained two or three exons, which differentiated them from mRNAs (Fig. 3c). Moreover, there was considerable diversity in the distribution of mRNA and lncRNA lengths (Fig. 3b). Furthermore, the expression level of most lncRNAs was significantly lower than that of mRNAs (Fig. 3d).

### Differentially expressed lncRNAs at various infection stages

**Fig. 4:**
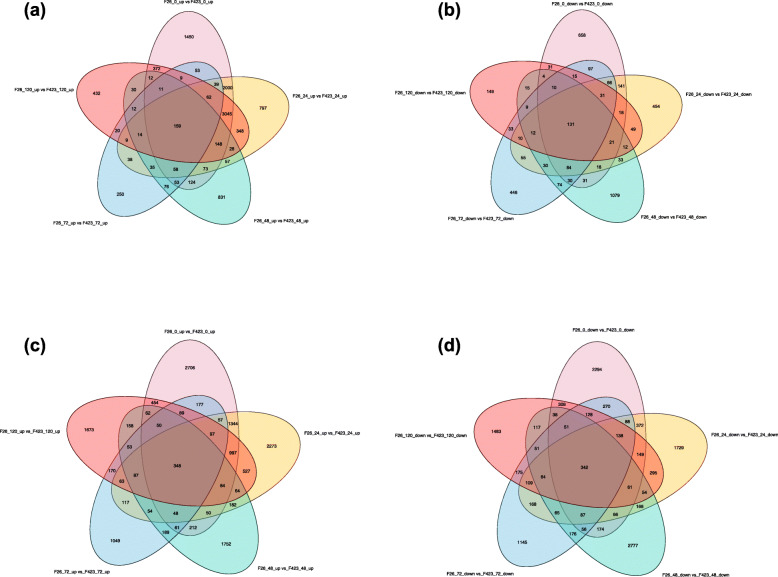
Gene expression profiles and number of differentially expressed genes for the disease-susceptible F423 walnut fruits and the disease-resistant F26 walnut fruits. The Venn diagram presents the (**a** and **c**) upregulated and (**b** and **d**) downregulated lncRNAs and mRNAs among five comparison groups (F26_0 vs F423_0, F26_24 vs F423_24, F26_48 vs F423_48,F26_72 vs F423_72, and F26_120 vs F423_120)

The lncRNAs that were differentially expressed between the disease-susceptible F423 fruits and the disease-resistant F26 fruits at different *C*. *gloeosporioides* infection stages were analyzed. Compared with F423, a total of 14,525 DELs were identified, including 10,645 up-regulated lncRNAs and 3846 down-regulated lncRNAs in F26. The number of upregulated and downregulated lncRNAs in the various comparisons were respectively as follows: 7668 and 1386 in the F26_0hpi vs F423_0hpi comparison; 6910 and 1165 in the F26_24hpi vs F423_24hpi comparison; 1721 and 1593 in the F26_48hpi vs F423_48hpi comparison; 898 and 1133 in the F26_72 hpi vs F423_72 hpi comparison; and 4711 and 550 in the F26_120 hpi vs F423_120 hpi comparison (Fig. 4a, b) (Additional file 6: Table S6). Additionally, compared with F423, a total of 34,007 differentially expressed mRNAs were identified, including 15,247 upregulated mRNAs and 13,198 downregulated mRNAs in F26. the number of upregulated and downregulated mRNAs in the various comparisons were respectively as follows: 6836 and 4622 in the F26_0 hpi vs F423_0 hpi comparison; 6392 and 3955 in the F26_24 hpi vs F423_24 hpi comparison; 3454 and 4347 in the F26_48 hpi vs F423_48 hpi comparison; 2709 and 3113 in the F26_72 hpi vs F423_72 hpi comparison; and 4976 and 3563 in the F26_120 hpi vs F423_120 hpi comparison (Fig. 4c, d) (Additional file 7: Table S7). These results revealed the similarities in the expression of lncRNAs and mRNAs. And the number of upregulated lncRNAs and mRNAs in F26 compared to in F423 was significantly higher at the early stages of *C. gloeosporioides* infection.

### Identification of co-expressed lncRNA modules

**Fig. 5:**
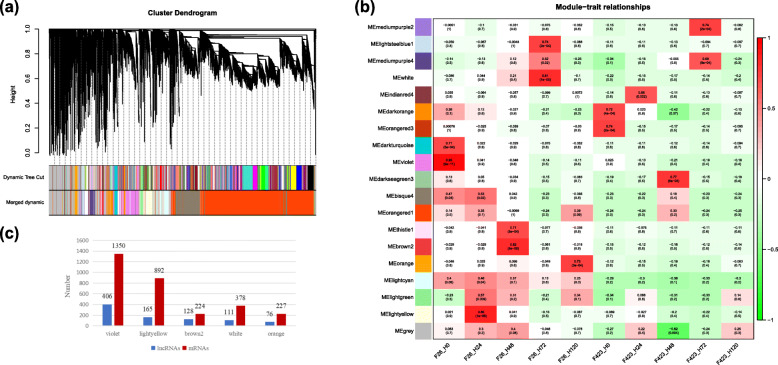
Weighted gene co-expression network analysis (WGCNA) of lncRNAs in all samples. **a** Hierarchical cluster tree presenting 19 modules of co-expressed lncRNAs. Each of the 10,645 lncRNAs is represented by a leaf in the tree, with each of the 19 modules presented as a major tree branch. The lower panel provides the modules in distinct colors. **b** Heatmaps indicating the correlation of module eigengenes at various infection stages. The Pearson correlation coefficients of each module at various stages are provided and colored according to the score. **c** The number of lncRNAs and mRNAs in five significant modules

To identify the hub lncRNAs and predict their potential target genes in trans-regulatory relationships, a weighted gene co-expression network analysis (WGCNA) was used to generate a correlation matrix of the expression levels of 10,645 upregulated lncRNAs and 15,247 upregulated mRNAs. A total of 19 expression modules were screened (Fig. 5a) (Additional file 8: Table_S8). The relationships between modules and the resistance traits of the walnut fruit bracts were analyzed and four significantly correlated modules (|r| ≥ 0.8) were identified. The MEviolet module was correlated with F26_0hpi (r = 0.95, *p* = 9e− 11), which contains 406 lncRNAs and 1350 mRNAs. The MElightyellow module was correlated with F26_24hpi (r = 0.86, *p* = 1e− 06), which contains 165 lncRNAs and 892 mRNAs. The MEbrown2 module was correlated with F26_48hpi (r = 0.82, *p* = 8e− 0.86), which contains 128 lncRNAs and 224 mRNAs. The MEwhite module was correlated with F26_72hpi (r = 0.81, p = 1e− 05), which contains 111 lncRNAs and 378 mRNAs (Fig. 5c). Regarding F26_120 hpi, the rand *p* value for the MEorange module was 0.73 and 3e− 0.4, respectively. The highest r value (0.77) for F423 was calculated for the MEdarkseagreen module and F423_48hpi (Fig. 5b). And the MEorange module contains 76 lncRNAs and 227 mRNAs (Fig. 5c). These results suggested that lncRNAs are closely related to the disease resistance of walnut fruit bracts.

### Enrichment analysis of genes co-expressed with lncRNAs

**Fig. 6:**
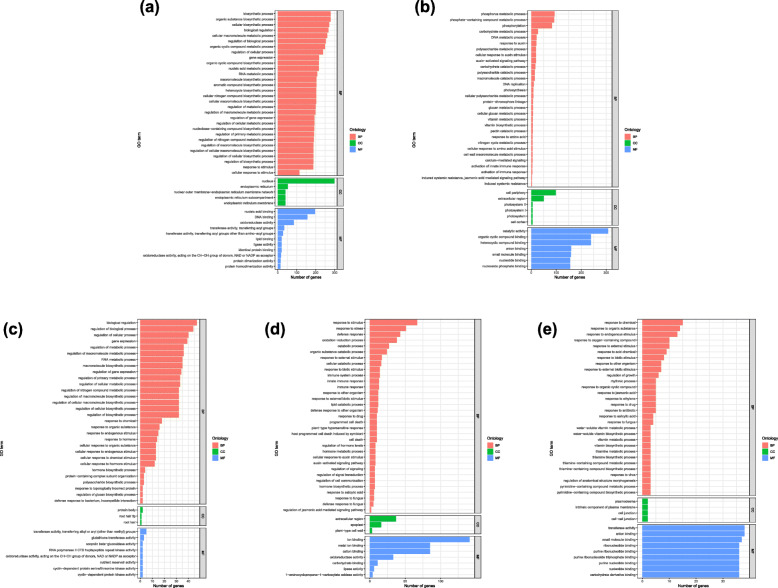
**a** Significantly over-represented GO terms in violet module for target genes. **b** Significantly over-represented GO terms in lightyellow module for target genes. **c** Significantly over-represented GO terms in brown2 module for target genes. **d** Significantly over-represented GO terms in white module for target genes. **e** Significantly over-represented GO terms in orange module for target genes

**Fig. 7:**
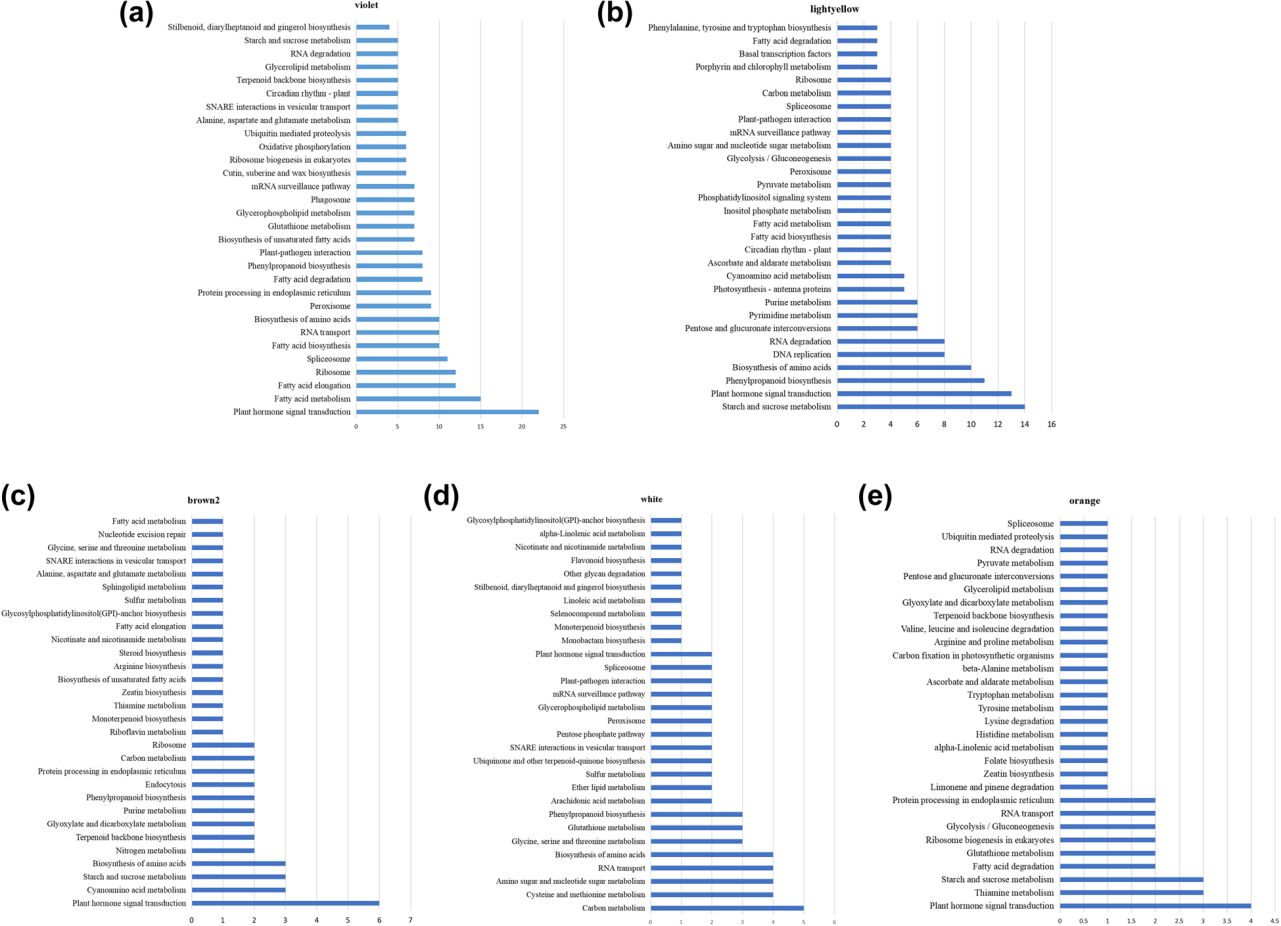
**a** Top 30 significantly enriched KEGG pathways in violet module for target genes. **b** Top 30 significantly enriched KEGG pathways in lightyellow module for target genes. **c** Top 30 significantly enriched KEGG pathways in brown2 module for target genes. **d** Top 30 significantly enriched KEGG pathways in white module for target genes. **e** Top 30 significantly enriched KEGG pathways in orange module for target genes

The GO and KEGG pathway databases were used to analyze the genes co-expressed with lncRNAs in each significant module and MEorange module. In the MEviolet module, a total of 208 GO terms were assigned, including 106, 8 and 94 GO terms in “biological process”, “cellular component” and “molecular functions”, respectively (Additional file 9:Table_S9). Among these enriched GO terms, most of them were related to biosynthesis and gene expression regulation, and the ones related to plant immunity were “response to stimulus”(GO:0050896) (187 genes) and “cellular response to stimulus”(GO:0051716) (114 genes) (Fig. 6a). In total, 104 enriched KEGG pathways were identified, of which 30 pathways were significantly enriched in this module (Additional file 10: Table_S10). The top 30 significantly enriched pathways for target genes are mentioned in Fig. 7a. “Plant hormone signal transduction” (ko04075) (22 genes), “Fatty acid metabolism” (ko01212) (15 genes), “Fatty acid elongation” (ko00062) (12 genes), “Ribosome” (ko03010) (12 genes), and “Spliceosome” (ko03040) (11 genes) were the most significant KEGG pathways.

In the MElightyellow module, a total of 164 GO terms were assigned, including 79, 16 and 69 GO terms in “biological process”, “cellular component” and “molecular functions”, respectively (Additional file 9: Table_S9). Among them, GO terms related to plant immunity included “activation of innate immune response” (GO: 0002218) (4 genes), “activation of immune response” (GO: 0002253) (4 genes), and “induced systemic resistance, jasmonic acid mediated signaling pathway” (GO: 0009864) (3 genes) (Fig. 6b). In total, 93 enriched KEGG pathways were identified, of which 30 pathways were significantly enriched in this module (Additional file 10: Table_S10). The top 30 significantly enriched pathways for target genes are mentioned in Fig. 7b. “Starch and sucrose metabolism” (ko00500) (14 genes), “Plant hormone signal transduction” (ko04075) (13 genes), “Phenylpropanoid biosynthesis” (ko00940) (11 genes), “Biosynthesis of amino acids” (ko01230) (10 genes), and “DNA replication” (ko03030) (8 genes) were the most significant KEGG pathways.

In the MEbrown2 module, a total of 126 GO terms were assigned, including 89, 5 and 32 GO terms in “biological process”, “cellular component” and “molecular functions”, respectively (Additional file 9: Table_S9). In addition to the terms related to biological metabolism and gene expression regulation, the items related to plant immunity “response to endogenous stimulus” (GO:0009719) (15 genes), “cellular response to endogenous stimulus” (GO:0071495) (13 genes) and “cellular response to hormone stimulus” (GO:0032870) (12 genes) were also enriched significantly (Fig. 6c). In total, 38 enriched KEGG pathways were identified, of which 30 pathways were significantly enriched in this module (Additional file 10: Table_S10). The top 30 significantly enriched pathways for target genes are mentioned in Fig. 7c. “Cyanoamino acid metabolism” (ko00460) (3 genes), “Plant hormone signal transduction” (ko04075) (6 genes), “Nitrogen metabolism” (ko00910) (2 genes), “Terpenoid backbone biosynthesis” (ko00900) (2 genes) were the most significant KEGG pathways.

In the MEwhite module, a total of 142 GO terms were assigned, including 95, 4 and 43 GO terms in “biological process”, “cellular component” and “molecular functions”, respectively (Additional file 9: Table_S9). Among the biological process category, the significantly over represented GO terms were “response to stimulus” (GO: 0050896) (67 genes), followed by “response to stress” (GO: 0006950) (51 genes) and “defense response” (GO: 0006952) (43 genes), which were all related to plant immunity. In addition, other terms related to plant immunity were also enriched, such as “immune system process” (GO:0002376) (14 genes), “response to biotic stimulus” (GO:0009607) (14 genes) and “innate immune response” (GO:0045087) (13 genes), etc. (Fig. 6d). In total, 54 enriched KEGG pathways were identified, of which 30 pathways were significantly enriched in this module (Additional file 10: Table_S10). The top 30 significantly enriched pathways for target genes are mentioned in Fig. 7d. “Carbon metabolism” (ko01200) (5 genes), “Cysteine and methionine metabolism” (ko00270) (4 genes), “Amino sugar and nucleotide sugar metabolism” (ko00520) (4 genes) were the most significant KEGG pathways.

In the MEorange module, a total of 128 GO terms were assigned, including 87, 8 and 33 GO terms in “biological process”, “cellular component” and “molecular functions”, respectively (Additional file 9: Table_S9). Among the biological process category, “response to organic substance” (GO: 0010033) (14 genes), “response to endogenous stimulus” (GO: 0009719) (13 genes), and “response to external stimulus” (GO: 0009605) (10 genes)etc., associated with plant immunity were significantly enriched (Fig. 6e). In total, 32 enriched KEGG pathways were identified, of which 30 pathways were significantly enriched in this module (Additional file 10: Table_S10). The top 30 significantly enriched pathways for target genes are mentioned in Fig. 7e. “Plant hormone signal transduction” (ko04075) (4 genes), “Thiamine metabolism” (ko00730) (3 genes), “Starch and sucrose metabolism” (ko00500) (3 genes) and “Fatty acid degradation” (ko00071) (2 genes) were the most significant KEGG pathways.

### Network analysis of hub lncRNAs

**Fig. 8:**
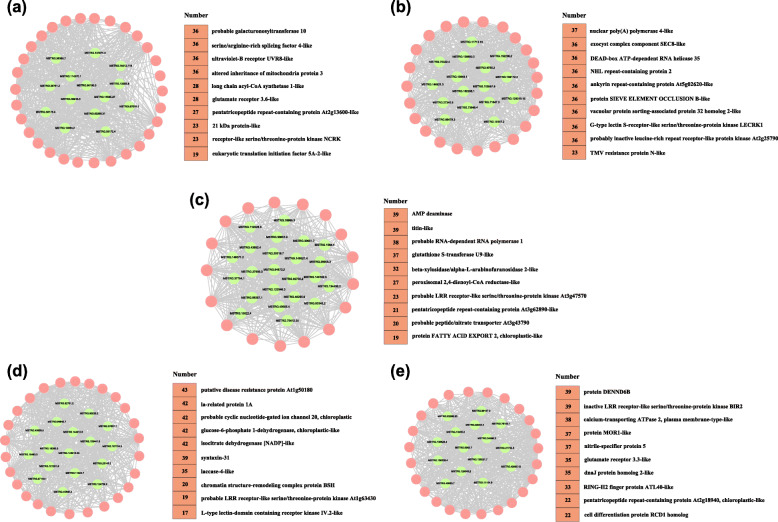
**a** Co-expression network associated with violet module. **b** Co-expression network associated with lightyellow module. **c** Co-expression network associated with brown2 module. **d** Co-expression network associated with white module. **e** Co-expression network associated with orange module. Red and green represent the target genes (mRNAs) and lncRNAs, respectively. Functional annotation of the target genes of the hub lncRNAs. Numbers represent the number of nodes

**Fig. 9:**
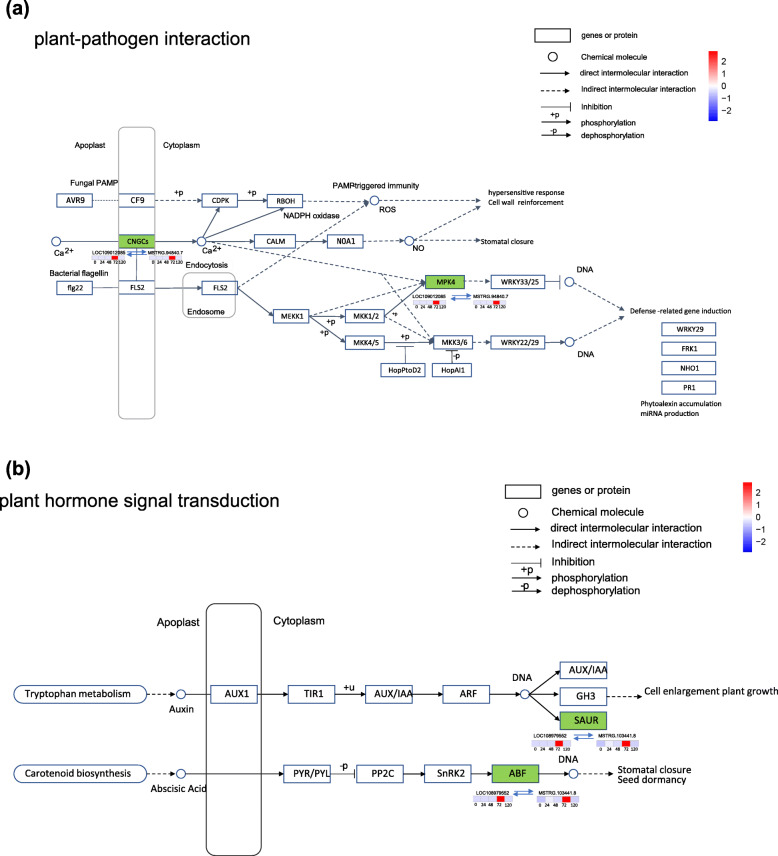
**a** MSTRG.94840.7 and the target gene LOC109012085 involved in plant-pathogen interaction pathway. **b** MSTRG.103441.8 and the target gene LOC108979552 involved in the plant hormone signal transduction pathway enriched by KEGG analysis

The hub lncRNAs are important for regulating the whole network. Therefore, we screened the 96 hub lncRNAs and 124 known target genes according to their weight value and connectivity in five modules (Additional file 11: Table_S11). In the MEviolet module, the 25 known target genes for 15 hub lncRNAs were found to be involved in multiple functions (Fig. 7a), such as probable galacturonosyl transferase 10 and ultraviolet-B receptor UVR8-like. In addition, target genes encoding receptor-like serine/threonine-protein kinase NCRK (XM_018958556.1) and eukaryotic translation initiation factor 5A-2-like (XM_018994862.1) are known resistance genes (Fig. 8a). In the MElightyellow module, 16 hub lncRNAs were generated and their 22 known target genes were involved in many functions (Fig. 8b). And the target genes encoding G-type lectin S-receptor-like serine/threonine-protein kinase LECRK1 (XM_018950446.1), probably inactive leucine-rich repeat receptor-like protein kinase At2g25790 (XM_018989953.1) and TMV resistance protein N-like (XM_018961957.1) were known resistance genes (Fig. 8b). In the MEbrown2 module, 24 hub lncRNAs and their 15 known target genes were generated (Fig. 8c), the target gene encoding probable LRR receptor-like serine/threonine-protein kinase At3g47570 (XM_018962714.1) was konwn resistance gene (Fig. 8c). In the MEwhite module, 23 hub lncRNAs were generated and their 38 known target genes were involved in many functions (Fig. 8d). The target genes encoding putative disease resistance protein At1g50180 (XM_018965430.1), probable LRR receptor-like serine/threonine-protein kinase At1g63430 (XM_018973294.1) and L-type lectin-domain containing receptor kinase IV.2-like (XM_018954279.1) were konwn resistance genes (Fig. 8d). In the MEorange module, 18 hub lncRNAs were generated and their 24 known target genes were involved in many functions (Fig. 8e). And the target gene encoding the inactive LRR receptor-like serine / threonine-protein kinase BIR2 (XM_018967526.1) was konwn resistance gene (Fig. 8e). All disease resistance genes in walnut are listed in Additional file 12: Table_S12. These results suggested that lncRNAs may participate in the resistance of walnut bracts to *C. gloeosporioides* by acting on their target genes. Based on the enrichment results of KEGG, we predicted the possible pathway of hub lncRNAs (Additional file 13: Table_S13). Most of the hub lncRNAs and its target genes in the five modules are enriched in the pathways of material metabolism and biosynthesis. In the white module, the function of hub lncRNA pathway map showed that cyclicnucleotide-gated channels and MPK4, the target genes of lncRNA MSTRG.94840.7,were upregulated at 72hpi, which were enriched in “plant pathogen interactions” pathway (Fig. 9a). The target genes (SAUR and ABF) of lncRNA103441.8 were involved in “plant hormone signal transduction” pathway,which may be related to plant immunity (Fig. 9b).

### Validation of hub lncRNAs and target genes

**Fig. 10:**
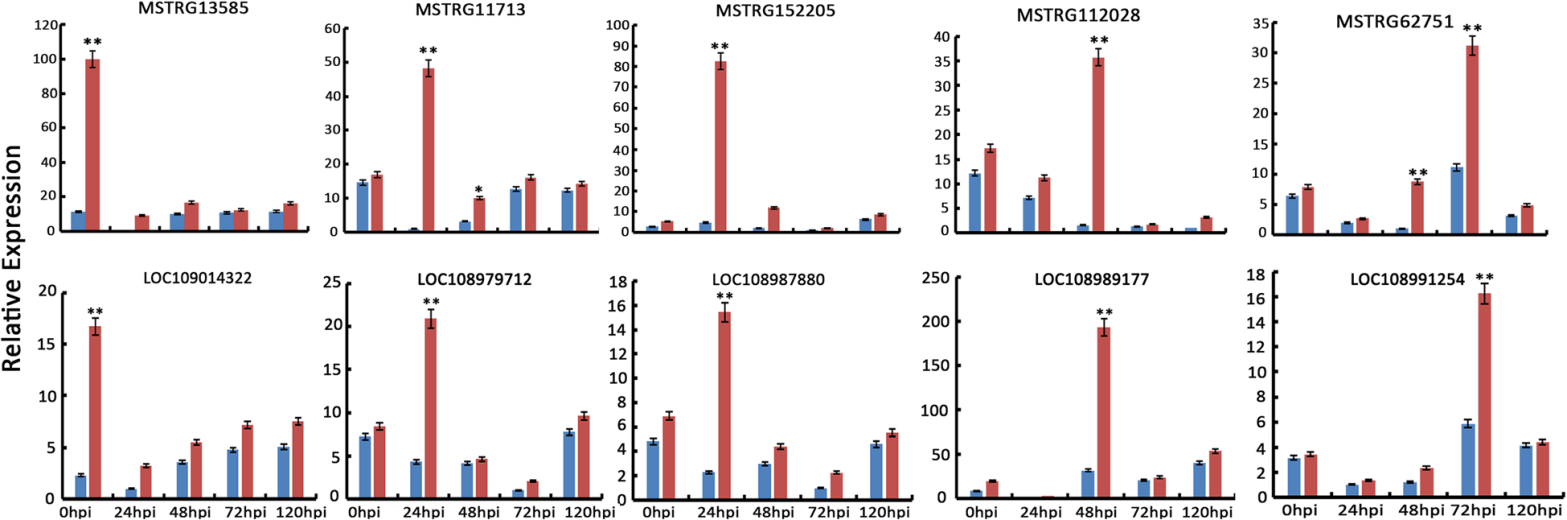
Validation of selected lncRNAs and mRNAs in a quantitative PCR assay. Blue and red represent the F423 and F26 samples, respectively. Expression data were normalized against the data for the18S rRNA housekeeping gene and are presented as themean ± standard error; *p < 0.05, **p < 0.01

We randomly selected 5 hub lncRNAs and 5 target genes for qRT-PCR analysis with the aim to validate the expression profiles between F26 and F423 obtained by RNA-Seq. The list of hub lncRNAs specific primers used for qRT-PCR analysis is listed in Additional file 14: Table_S14. The hub lncRNAs selected for qRT-PCR confirmation were MSTRG.13585.8, MSTRG.152205.1, MSTRG.11713.16, MSTRG.112028.8, and MSTRG.62751.2, the target genes were related to probable galacturonosyl transferase 10 (LOC109014322), G-type lectin S-receptor-like serine / threonine-protein kinase LECRK1(LOC108979712), NHL repeat-containing (LOC108987880), probable LRR receptor-like serine/threonine-protein kinase At3g47570 (LOC108989177), and putative disease resistance protein At1g50180 (LOC108991254). The qRT-PCR analysis showed that the expression of MSTRG13585 and LOC109014322 peaked at 0hpi, MSTRG11713, MSTRG152205, LOC108979712 and LOC108987880 at 24hpi, MSTRG112028 and LOC108989177 at 48hpi, MSTRG62751 and LOC108991254 at 72hpi (Fig. 10), which were consistent with the RNA-seq data (Additional file 15: Table_S15), with similar trends observed for the hub lncRNAs and their target genes.

## Discussion

In previous studies, lncRNAs were identified and analyzed in various biological processes important for seed development [31], photomorphogenesis [32], fruit development [33, 34], and biotic and abiotic stress responses [22, 35]. Additionally, there has been substantial research on the role of lncRNAs in plant–pathogen interactions. In *A. thaliana*, lncRNAs reportedly enhance the resistance to *Pseudomonas syringae* pv. *tomato* DC3000 by promoting *PR1* expression [36]. In tomato, lncRNA23468 functions as a ceRNA that modulates NBS-LRR gene expression by mimicking the target of miR482b, thereby increasing the resistance to *P. infestans* [37]. Walnut anthracnose has been responsible for the premature fruit drop and yield losses that have adversely affected walnut production in China [13]. In this study, we investigated the role of lncRNAs in the resistance of walnut fruit bracts to anthracnose based on sequence analyses. Walnut anthracnose is caused by *C. gloeosporioides*, which completes its infection process as a hemibiotroph [10, 38]. First, conidia germinate to generate appressoria, which produce invasion pegs that initiate the infection into susceptible plants. The primary mycelium produced in plant cells exists as a biotroph, after which the secondary mycelium produced in the infected site switches to necrotrophic growth [39, 40]. We previously determined that the *C. gloeosporioides* life cycle in walnut tissue involves attachment at 24hpi, biotrophy at 48hpi, and necrotrophy at 72hpi (data unpublished). In this study, RNA-seq was performed to build the lncRNA and mRNA profiles of the walnut fruit bract tissue at 0 hpi, infected tissue at 24, 48, and 72 hpi, and distal uninoculated tissue at 120 hpi. A total of 58,369 mRNAs and 22,336 lncRNAs were identified, including 3933 known lncRNAs and 18,403 unknown lncRNAs. Consistent with the results of similar studies on other organisms, the identified putative lncRNA had fewer exons, shorter transcripts, and lower expression levels than protein-coding genes [41, 42].

The release of walnut reference genome [1], enabled the study of walnut genetics at a genome-wide scale. Based on the reference genome, the whole-genome resequencing [43], the development of high-density genotyping tools [44], and the genetic dissection of important agronomical traits in walnut [45] have been completed. The development of bioinformatic analysis technology has enabled researchers to reveal that lncRNA functions and characteristics are far more complex than previously thought [16]. A recent comparative transcriptome analysis between wild-type and *WRKY1*-overexpressing tomato plants revealed 199 lncRNAs (DELs) and indicated that many of the lncRNA target genes that are likely affected by WRKY1 and associated with the resistance of tomato to *P. infestans* are involved in the response to biotic stimulus (GO:0009607) and plant-pathogen interaction (KO4626) [26]. In another recent study, 4594 putative lncRNAs were identified in comprehensive dynamic lncRNA expression networks under heat stress conditions. Co-expression networks revealing the interactions among the differentially expressed lncRNAs, mRNAs, and microRNAs indicated that several phytohormone pathways are associated with heat tolerance, including salicylic acid and brassinosteroid pathways [21]. In the current study, we obtained 10,645 upregulated lncRNAs and 15,247 upregulated mRNAs among the five comparisons (F26_0hpi vs F423_0hpi, F26_24 hpi vs F423_24 hpi, F26_48 hpi vs F423_48 hpi, F26_72 hpi vs F423_72 hpi, and F26_120 hpi vs F423_120 hpi). The number of up-regulated lncRNAs and mRNAs in the F26 vs F423 was significantly higher at the early stages of *C. gloeosporioides* infection.

The functions of lncRNAs cannot currently be inferred from their sequence or structure, but lncRNAs can function in trans mode to target gene loci distant from where the lncRNAs are transcribed [46]. In F26, a total of 5 modules related to disease resistance were obtained by WGCNA during the infection of *C. gloeosporioides*. Many target genes of lncRNAs in these modules are enriched in plant immune related items and pathways, such as “activation of innate immune response”, “activation of immune response” in MElightyellow module, “defense response to bacterium, incompatible interaction” in MEbrown2 module, “defense response” and “immune system process” in MEwhite module. These results suggest that these genes may play important roles in the process of resistance to *C. gloeosporioides* of walnut fruit bracts. Phytohormones are known to be important in the regulation of defense responses in plants [47–49]. Plants can exhibit systemic acquired resistance through the salicylic acid (SA) / jasmonic acid (JA)-mediated signaling network [50–53]. In our study, a total of 32 genes were identified in the significantly enriched KEGG pathway “Plant hormone signal transduction”. Meanwhile, there are 3 and 5 genes enriched in “jasmonic acid mediated signaling pathway”and “response to jasmonic acid” respectively. We also showed that some genes were enriched in “auxin-activated signaling pathway” and “cellular response to auxin stimulus” at 24 hpi. Therefore, auxin may play a role in the resistance of walnut bracts to *C. gloeosporioides*. In addition, our result showed that the phenylpropanoid biosynthesis was one of the most significantly enriched pathways in the process of resistance to *C. gloeosporioides* of walnut fruit bracts. In this pathway, phenylalanine ammonium lyase (PAL) is the key regulatory enzyme in altering the biosynthesis and accumulation of flavonoids and lignin [54]. Lignin plays a structural role in the secondary cell walls formation [55], and flavonoids mediate plants against UV radiation and act as a visual signal for attracting pollinators [56, 57]. In *Caragana korshinskii*, *C. korshinskii* adjusts its phenylpropanoid biosynthesis process to water-deficit environments and activates PAL by drought stress [58].

During long-term evolutionary interactions with plants, several pathogens successfully cause effector-triggered susceptibility response (ETS) by producing a number of effectors. Simultaneously, plants have evolved R genes that recognize these effectors and function through highly specific interactions between effectors and their corresponding nucleotide-binding site and leucine-rich repeat (NB-LRR) class receptors [59]. In tomato, lncRNA23468 reportedly increases the expression of the NBS-LRR target genes (encoding R proteins), resulting in enhanced resistance to *P.infestans* [37]. In the current study, we detected 10 *R* genes among the target genes of 96 hub lncRNAs. During the infection of *C. gloeosporioides* on the walnut fruit bracts, the results of RNA-seq showed that the expression of *R* genes (XM_018950446.1, XM_018989953.1 and XM_018961957.1 in MElightyellow module, XM_018962714.1 in MEbrown2 module, XM_018965430.1, XM_018973294.1 and XM_018954279.1 in MEwhite module) were up-regulated at 24hpi, 48hpi and 72hpi respectively, and expression of the highly connected lncRNAs (MSTRG.11713.16, MSTRG.146621.3 and MSTRG.136680.2 in MElightyellow module, MSTRG.123346.3 in MEbrown2 module, MSTRG.94840.7, MSTRG.18285.3 and MSTRG.45846.2 in MEwhite module) had the same trends (Additional file 14 Table_S14). These findings imply that lncRNAs may help mediate the disease resistance of walnut fruit bracts through the target *R* genes. The specific interaction between lncRNAs and *R* gene needs further verification. The expression levels of five hub lncRNAs (MSTRG13585, MSTRG11713, MSTRG152205, MSTRG112028, and MSTRG62751) and their target genes were further confirmed by qPCR, the results of which were consistent with the RNA-seq data. The data presented here provides researchers with the biological basis for future investigations of the mechanism underlying the disease resistance of walnut fruit bracts.

## Conclusions

In this study we generated the expression profile of lncRNA in anthracnose-resistant F26 and anthracnose-susceptible F423 at five times. Compared with F423, a total of 14,525 DELs were identified, including 10,645 upregulated lncRNAs and 3846 downregulated lncRNAs in F26. Bioinformatic analysis showed that the target genes of upregulated lncRNAs were enriched in immune-related processes, plant hormone signal transduction, phenylpropanoid biosynthesis and other pathways during the infection of *C. gloeosporioides*. Hub lncRNAs with high connectivity to disease resistant genes were predicted. These results contribute to our understanding of the potential mechanism by which lncRNAs involved in *C. gloeosporioides* resistance and will facilitate the functional verification of the lncRNA in the future.

## Methods

### Plant materials and fungal isolates

The scions of walnut seedling tree B26 was provided by walnut specialized farmers’ cooperative of Dongliugang village, Baishi Town, Wenshang County, Shandong Province, China (35°46′56.2″N, 116°40′30.8″E). The 4–23 walnut tree was from F1 progeny of an intraspecific cross between walnut cultivar ‘Yuan Lin’ (susceptible to anthracnose) × ‘Qing Lin’ (resistant to anthracnose) which was carried out by ourselves in 2002. The plant materials were conserved by patch budding onto walnut seedling rootstock at the Forestry Experimental Station of Shandong Agricultural University, Tai’an, Shandong Province, China (36°10′ 19.2″N, 117°09′ 1.3″E) in late May 2009. In 2015–2017, we evaluated the anthracnose resistance of each plant for three consecutive years followed by previously described [8, 14], and it was found that B26 clone was highly resistant to anthracnose in fruit bract, and the 4–23 clone was highly susceptible to anthracnose in fruit bract. The fruits of B26 clone (i.e., F26) and 4–23 clone (i.e., F423) were used as experimental materials. The voucher specimen of F26 and F423 had been deposited to our lab but not to any publicly available herbarium. We didn’t use wild plants in this study and according to national and local legislation, no specific permission was required to collect these plants. *C.gloeosporioides*m9 isolates (GenBank ID: GU597322) used in this study were maintained by our group.

### Fungal pathogen inoculation of walnut fruits

*Colletotrichum gloeosporioides* was cultivated on potato dextrose agar medium for 5–7 days at 28 °C.To prepare conidial suspensions, the colonies were washed with sterile distilled water containing 0.05% (v/v) Tween 80, passed through a filter (40–100 μm pores), quantified with a hemocytometer, and diluted with sterile distilled water to 105–106 conidia/ml [0.001% (v/v) Tween 80 final concentration]. Healthy fruits from the east-, south-, and west-facing parts of each tree were collected in mid-June and disinfected with 0.6% sodium hypochlorite and rinsed with sterile water. The punch inoculation of the detached walnut fruits was completed as previously described [8]. Based on anatomical changes to the infected walnut fruit bract, samples of the inoculation site were collected at 0, 24, 48, and 72 hpi, 0 hpi as a control. Additionally, distal uninoculated tissue was collected at 120 hpi. Take two independent samples as biological replicates at each infection time (Additional file 1: Table S1). All samples were flash-frozen in liquid nitrogen and stored at − 80 °C until analyzed.

### Determination of physiological and biochemical data

The activity of CAT, Chitinase, SOD and contents of H_2_O_2_, salicylic acid and jasmonic acid at five time points of F26 and F423 were determined according to the instructions on the kit. Each sample was repeated three times. The CAT, Chitinase and SOD activity levels were measured and performed according to kit instructions (Solarbio, cat. No. BC0820) and detected by TU-1901 UV Spectrophotometer (Beijing Purkinje General Instrument Co.,Ltd., Beijing). The content of H_2_O_2_, salicylic acid and jasmonic were detected by the Solebao kit (Solarbio, cat. No. BC3595) with microdetermination.

### RNA extraction, library construction, and sequencing

Total RNA was extracted from F423 and F26 samples with the Thermo Gene JET Plant RNA Purification Mini Kit (Thermo Fisher Scientific Inc., USA). The purity and concentration of the extracted RNA were determined with the NanoDrop2000 spectrophotometer (Thermo Fisher Scientific Inc.) (OD_260/280_ ≥ 1.8, OD_260/230_ ≥ 1.5, and concentration > 40 ng/μl). The RNA integrity was assessed by agarose gel electrophoresis. Ribosomal RNA was removed with the Ribo-Zero™ Magnetic Kit (Epicentre) and the remaining RNA (polyA+ and polyA−) was recovered. The RNA was randomly fragmented to approximately 200-bp sequences in Fragmentation Buffer (Thermo Fisher Scientific Inc.) and then used as the template to synthesize first-strand cDNA with random hexamers. The second cDNA strand was synthesized with dNTPs, RNaseH, and DNA polymerase I. The overhanging ends were filled in with T4 DNA polymerase and Klenow DNA polymerase to generate blunt ends, after which the A base was added to the 3′ end and the fragment was ligated to a linker. The AMPureXP beads were used for selecting fragments. The second cDNA strand containing U was degraded with the USER enzyme, after which a sequencing library was obtained by PCR amplification. A total of 20 sequencing libraries were constructed. The Qubit 2.0 DNA Broad Range Assay (Invitrogen, USA) was used for a preliminary quantification. The sequencing library inserts were detected with the Agilent 2100 Bioanalyzer. Finally, the effective library concentrations (> 2 nM) were accurately quantified by qPCR. Paired-end sequencing (2 × 150 bp) was completed in KeGene Science & Technology Co. Ltd. (Shandong, China) with an Illumina HiSeq 4000 platform.

### Read mapping and transcriptome assembly

The quality of the raw sequencing data was checked with FASTQC (http://www.bioinformatics.babraham.ac.uk/projects/fastqc/). Adapters and low-quality tags in the raw data were eliminated. Ribosomal RNA data were also removed. The remaining clean reads for the 20 cDNA libraries were combined and mapped to the *J. regia* genome sequence (https://www.ncbi.nlm. nih.gov/genome/? term = Juglans% 20 regia) with the HISAT program (version 0.11.5) (parameter setting: -rna-strandness RF) [60]. To construct transcriptomes, the mapped reads were assembled with StringTie (version 1.3.1) [61]. After combining the StringTie results for each sample with StringTie-Merge, the read counts were calculated for transcripts with bedtools (version 2.27.1) (bedtools.readthedocs.org /en/latest/#) [62].

### Identification of lncRNAs

To obtain the potential long non-coding RNAs, based on all the assembled transcripts, we have firstly excluded the known transcripts according to the class code “=”. Then the remaining transcripts were used to remove the potential protein coding transcripts, miRNA-like, and other transcript types via blasting against the database of Rfam, Refseq, Uniprot, miRbase, and Pfam. Finally, the remaining transcripts were employed for coding potential prediction by using FEELnc tool. First, the FEELnc filter was used to remove short transcripts (default 200 nt) and assess single-exon transcripts [63]. The FEELnc codpot predictors were used to calculate a coding potential score. The assembled sequences were used for reconstructing the transcriptome. Finally, RNAs longer than 200 nt and derived from ≥2 exons, with an ORF coverage < 50% and a potential coding score < 0.5 were designated as lncRNAs [64].

### Classification of lncRNAs

The lncRNAs were analyzed regarding their corresponding positions in the reference genome and the positional relationships between lncRNAs and partner RNAs based on 10,000–100,000 fragments. The lncRNAs were then divided into genic lncRNAs (overlapping partner RNAs) and intergenic lncRNAs (lincRNAs). The genic lncRNAs were further divided as overlapping, containing, or nested subtypes. Intergenic lncRNAs were divided as divergent, convergent, and same strand subtypes.

### Analysis of differential expression patterns

Genes differentially expressed between the disease-resistant and susceptible fruits at five infection stages were analyzed with DESeq2 (version 1.22.1) [65]. After assessing the significance of any differences, the genes with a *p* value ≤0.05 and a |log_2_foldchange| ≥ 1 were designated as differentially expressed genes. The principal component analyses (PCA) of F26 and F423 were constructed using the prcomp() function shipped with the base R installation. The PCA result was visualized using the ggplot2 package in R.

### Quantitative real-time PCR

Total RNA samples extracted from walnut fruitsat individual infection stages were analyzed by qPCR. Briefly, first-strand cDNA was obtained with the TransScript One-Step gDNA Removal and cDNA Synthesis SuperMixfor qPCR (Transgen, China). The lncRNA expression level was quantified with the TransStart Tip Green qPCR SuperMix (Transgen) and the CFX Connect Real-TimeSystem (Bio-Rad). The qPCR program was as follows: 95 °C for 30 s; 40 cycles of 95 °C for 10 s and 60 °C for 30 s. For a melting curve analysis, the temperature was increased from 70 °C to 95 °C (0.5 °C/5 s). All samples were analyzed in triplicate. The 18S rRNA gene was used as a housekeeping gene. The cycle threshold (Ct) 2^-ΔΔCt^ method (Software IQ5 2.0) [66] was used for the relative quantification of mRNAs. The primers used for RT-qPCR were designed with Beacon Designer 7 software and were synthesized by Sangon Biotech (Shanghai, China; Supplementary Table S14).

### Prediction of lncRNA functions based on co-expression

Co-expression modules were generated with the WGCNA package (version 1.67) as previously described [67] (http://lab.genetics.ucla.edu/horvath/Coexpression Network/). The lncRNAs and mRNAs that were not detected in at least one infection stage were not considered. In this analysis, the soft thresholding power was set to 12, after which the adjacency function was used to construct the adjacency matrix. A topological overlap measure map was constructed based on the adjacency matrix to calculate the similarity matrix of the lncRNA and mRNA expression between different nodes. The lncRNAs and mRNAs were hierarchically clustered based on the algorithm. To generate a number of clusters, modules were defined after eliminating or combining branches. The co-expression module dynamic shear tree parameters were determined as described by Gerttula [68]. The minimum number of genes was set to 30, the split sensitivity (deep Split) was set to 2, and the other settings were software default parameters. The module was related to the trait, and the correlation matrix between the module and the trait was calculated. The module with the highest correlation coefficient and the smallest p value was designated as the module most relevant to the trait. In this study, a significantly correlated module was identified based on a correlation coefficient (r) ≥ 0.8 [64] and *p* < 0.05. The co-expression networks of lncRNAs and hub lncRNAs in highly correlated modules were generated with the Cytoscape software (version 3.7.1) [69].

### Functional enrichment analysis

The genes targeted by lncRNAs were functionally annotated based on the GO and KEGG pathway (http://www.genome.jp/kegg/) databases. The KOBAS program (version 2.0) was used to determine the significantly enriched KEGG pathways among the target genes [70]. According to the operation requirements of KOBAS 2.0, All data files were written with a parser. The gene-term mapping can be retrieved by parsing the raw data files for each pathway. The gene annotation and gene-ID relations were retrieved from KEGG Genes and BioMart. We mapped the genes in all databases to KEGG GENES and KEGG ORTHOLOGY (KO). The gene-pathway and is stored in our backend SQL relational database. The FASTA protein sequence files were preprocessed for BLAST [71].

## Supplementary Information

**Additional file 1: Table S1.** Detailed information on ten walnut samples for RNA-seq.

**Additional file 2: Table S2.** Summary of the sequencing information.

**Additional file 3: Table S3.**The expression level (reads counts) for each lncRNA genes detected in our study.

**Additional file 4: Table S4.** The expression level (reads counts) for each mRNA genes detected in our study.

**Additional file 5: Table S5.** TheClassification results of lncRNAs.

**Additional file 6: Table S6.** All theupregulated and downregulatedlncRNAsamong the five stages.

**Additional file 7: Table S7.** All theupregulated and downregulatedmRNAsamong the five stages.

**Additional file 8: Table S8.** The genes information of different modules in the WGCNA.

**Additional file 9: Table S9.** List of significantly enriched GO terms in five modules.

**Additional file 10: Table S10.** Summary of KEGG annotations for five modules.

**Additional file 11: Table S11.** All hub lncRNAs of five significant modules. Genes from the five modules in the F26.

**Additional file 12: Table S12.** All the disease resistance genes in walnut.

**Additional file 13: Table S13.** The function of hub lncRNAs pathway.

**Additional file 14: Table S14.** Thesequences of quantitative real time PCR primers.

**Additional file 15: Table S15.** Expression trend of disease resistance genes and hub lncRNAs.

## Data Availability

The data sets are included within the article and its Additional files. The raw sequencing data were deposited in NCBI Sequence Read Archive under the accession number GSE147083. (https://www.ncbi.nlm.nih.gov/geo/query/acc.cgi?acc=GSE147083).

## Abbreviations

LncRNAs: Long non-coding RNAs
DELs: Different expressed lncRNAs
WGCNA: Weighted Gene Coexpression Network Analysis
hpi: Hours post inoculation
eTM: Endogenous target mimics
ceRNA: Competing endogenous RNA
GO: Gene Ontology
KEGG: Kyoto Encyclopedia of Genes and Genomes
ETS: Effector-triggered susceptibility response; NB-LRR: nucleotide-bindingsite and leucine-rich repeat
ROS: Reactive oxygen species
SA: Salicylic acid
JA: Jasmonic acid
